# A biogenic amine and a neuropeptide act identically: tyramine signals through calcium in *Drosophila* tubule stellate cells

**DOI:** 10.1098/rspb.2012.2943

**Published:** 2013-04-22

**Authors:** Pablo Cabrero, Laura Richmond, Michael Nitabach, Shireen A. Davies, Julian A. T. Dow

**Affiliations:** 1 Institute of Molecular, Cell and Systems Biology, College of Medical, Veterinary and Life Sciences, University of Glasgow, Glasgow G12 8QQ, UK; 2 Department of Cellular and Molecular Physiology, Department of Genetics, Program in Cellular Neuroscience, Neurodegeneration and Repair, Yale School of Medicine, 333 Cedar Street, New Haven, CT 06520, USA

**Keywords:** Malpighian tubule, insect physiology, ion transport, endocrinology, aequorin, calcium

## Abstract

Insect osmoregulation is subject to highly sophisticated endocrine control. In *Drosophila*, both *Drosophila* kinin and tyramine act on the Malpighian (renal) tubule stellate cell to activate chloride shunt conductance, and so increase the fluid production rate. *Drosophila* kinin is known to act through intracellular calcium, but the mode of action of tyramine is not known. Here, we used a transgenically encoded GFP::apoaequorin translational fusion, targeted to either principal or stellate cells under GAL4/UAS control, to demonstrate that tyramine indeed acts to raise calcium in stellate, but not principal cells. Furthermore, the EC(50) tyramine concentration for half-maximal activation of the intracellular calcium signal is the same as that calculated from previously published data on tyramine-induced increase in chloride flux. In addition, tyramine signalling to calcium is markedly reduced in mutants of *NorpA* (a phospholipase C) and *itpr*, the inositol trisphosphate receptor gene, which we have previously shown to be necessary for *Drosophila* kinin signalling. Therefore, tyramine and *Drosophila* kinin signals converge on phospholipase C, and thence on intracellular calcium; and both act to increase chloride shunt conductance by signalling through *itpr*. To test this model, we co-applied tyramine and *Drosophila* kinin, and showed that the calcium signals were neither additive nor synergistic. The two signalling pathways thus represent parallel, independent mechanisms for distinct tissues (nervous and epithelial) to control the same aspect of renal function.

## 1. Introduction

Insect Malpighian tubules play key roles in ion transport and excretion [1], immune function [2,3] and xenobiotic detoxification [4,5]. Because of these multiple roles, they are also important both in sensing and in mounting a homeostatic response to stress [6–12]. They even show positional and gender-specific asymmetry in function [13]. Their neuroendocrine control is appropriately sophisticated, and well reviewed elsewhere [1,14,15].

The *Drosophila melanogaster* tubule is an excellent model for insect tubules, particularly of Diptera, which segregate their transport function into two specialized cell types [16]. Active cation transport is energized by an apical plasma membrane H^+^ V-ATPase, which drives alkali metal–proton exchange to produce a net transport of potassium or sodium, so increasing the transepithelial potential (TEP) [1]. Several neuropeptides have been linked to activation of the principal cell: the diuretic hormones DH_31_ [17] and DH_44_ [18], which both act through cyclic AMP; CAPA [19], acting through calcium; and Nplp1-4, an ‘orphan’ peptide [20] that was recently shown to activate a receptor guanylate cyclase [10]. Activation of the principal cell alone produces a modest increase in fluid secretion, because the resting chloride conductance is relatively low.

Stellate cells are activated by *Drosophila* kinin, or Drosokinin (NSVVLGKKQRFHSWGamide) [21], a member of a neuropeptide family found in most insects [22,23], which signals through a canonical G-protein coupled receptor (GPCR) to raise intracellular calcium [24], and thence to rapidly increase the chloride shunt conductance, effectively removing the ‘brake’ on active cation pumping, resulting in a rapid collapse of TEP and concomitant increase in fluid secretion [25,26].

Recently, it has become clear that tyramine is a second agonist for the stellate cell [6,27,28]. Like *Drosophila* kinin, it signals through a canonical GPCR and acts to collapse the TEP, and so increase fluid secretion. It is thus of great interest to establish whether tyramine acts through intracellular calcium, and whether the *Drosophila* kinin and tyramine signals interact in any way. This is particularly straightforward to address in *Drosophila*, with ready availability of classical mutants, and powerful transgenics—indeed the first report of the use of a genetically encoded calcium sensor in animals was in *Drosophila* [29]. Here, as well as demonstrating that tyramine does indeed signal through intracellular calcium in only the stellate cells, we report the use of an improved calcium sensor in tubules that is based on a translational fusion of the two jellyfish photoproteins apoaequorin and green fluorescent protein (GFP), resulting in markedly improved sensitivity [30,31].

## 2. Material and methods

### (a) *Drosophila* maintenance

*Drosophila* were kept at 25°C, 12 : 12 h photoperiod and 45–55 per cent relative humidity, and raised on standard *Drosophila* medium, as described previously [32].

### (b) Generation of calcium reporter flies

We have previously described the use of quantitative reporters based on transgenic aequorin [29], as well as imaging reporters based on pericam [33]; here, we generated flies transgenic for a calcium reporter based on a translational fusion of GFP and apoaequorin, under control of the UAS control region (‘UAS-GFP::aeq’) by cloning a synthetic cDNA into the transformation vector pP{UAST} and germ-line transforming *Drosophila* according to standard protocols. As reported elsewhere, we found that such a reporter shows greatly increased stability and luminescence [30], allowing superior real-time recordings to be obtained with less tissue in each sample.

### (c) Real-time intracellular calcium assays

Assays were as described earlier [29]. Briefly, week-old adult flies were anaesthetised by chilling on ice for a few minutes, then tubules dissected in Schneider's culture medium (except as described below). Where reduced tyrosine or tyramine levels were required, tissues were dissected and assayed in standard *Drosophila* saline [32], which does not contain these compounds. Depending on the experiment, tubules expressed UAS-GFP::aeq, driven by GAL4 lines c42 (specific to principal cells in the main segment) or c724 (specific to stellate cells).

Tubules were incubated in the dark with coelenterazine to reconstitute active aequorin, then real-time luminescence measured in a Berthold luminometer. After establishing a stable baseline, tyramine or *Drosophila* kinin was applied through injectors, and response was followed up for a further period. At the end of the experiment, undischarged aequorin was measured by permeabilizing the cells with Triton X-100 in the presence of excess calcium. Instantaneous real-time calcium values throughout the experiment were then back-calculated with an in-house Perl routine, based on standard methods [34].

### (d) Statistics

Data are plotted as mean ± s.e.m. Where needed, data were compared using Student's *t*-test, taking *p* = 0.05 (two-tailed) as the critical value. For EC_50_ values, best fit was calculated by least-squares nonlinear fit (GraphPad Prism), and the resulting log(EC_50_) values compared with a *t*-test.

## 3. Results and discussion

**Figure 1.**
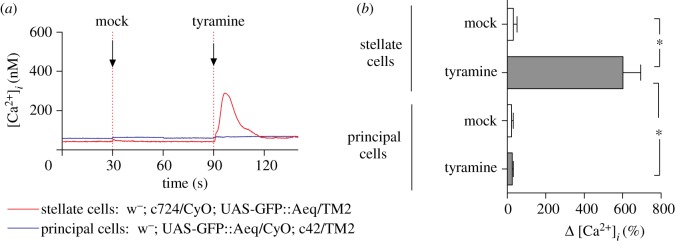
Tyramine acts to raise intracellular calcium in stellate, but not in principal cells. (*a*) Representative experiment, in which an apoaequorin::eGFP fusion was expressed in principal cells by crossing to the c42 GAL4 driver (blue), or only in stellate cells, by crossing to the c724 GAL4 driver (red). A mock injection before the addition of the secretagogue (at 5 × 10^−8^ M) allows any injection artefact to be estimated; in this case, it was negligible. (*b*) Summary of peak responses from three such experiments. Significant differences are marked with an asterisk.

The action of tyramine is to collapse the TEP across the tubule by rapidly increasing the chloride shunt conductance, and thus to stimulate KCl transport and fluid production [27]. These are the same actions ascribed to the neuropeptide *Drosophila* kinin, which has been shown to act to raise intracellular calcium only in stellate cells [24,35], implying that the chloride shunt conductance route is controlled by these cells. Consistent with this, the *Drosophila* kinin receptor is found in stellate cells in *Drosophila* [24], *Anopheles* [35] and *Aedes* [36]. Accordingly, tyramine was applied to tubules transgenic for the enhanced aequorin::GFP fusion, which provides a sensitive, real-time, absolute measurement of intracellular calcium (figure 1). When GFP::Aeq was driven in principal cells, no response to tyramine was seen; but when driven in stellate cells, a prominent, rapid calcium rise was observed, as previously documented for *Drosophila* kinin. Therefore, tyramine, like *Drosophila* kinin, acts to raise intracellular calcium in only stellate cells.

**Figure 2.**
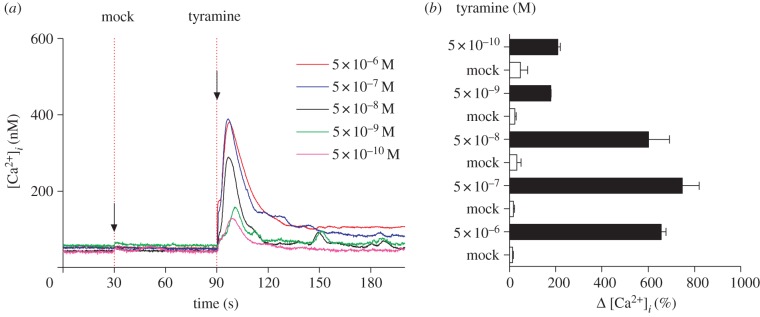
Concentration dependence of tyramine activation of intracellular calcium in stellate cells. (*a*) Typical responses to varying concentrations of tyramine, injected at 90 s. (*b*) Mean response across a range of concentrations, compared to corresponding mock injections (*n* = 3 except for *n* = 2 at 5×10^−10^ M).

**Figure 3.**
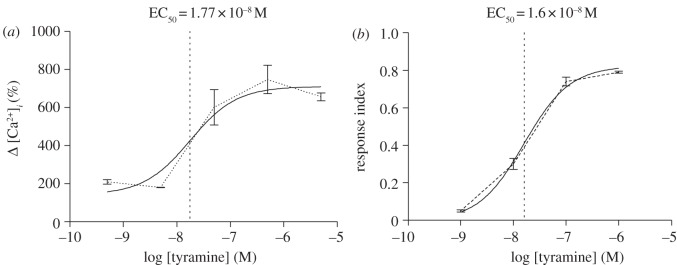
The EC_50_ for tyramine activation of stellate cell intracellular calcium matches that calculated for activation of chloride conductance. (*a*) Data from figure 2 were re-plotted as a standard semi-log dose–response curve, and a curve (solid line) fitted to the original data (dotted line) using GraphPad Prism. (*b*) Data were re-measured from fig. 2*c* of [4], and re-plotted as in (*a*).

The tyramine response was concentration-dependent (figure 2), with an EC_50_ of 1.77 × 10^−8^ M (figure 3*a*). To test whether this was relevant to the functional endpoint of elevated shunt conductance, this value was compared with the EC_50_ for chloride shunt conductance activation, assayed as a change in TEP [27]. No formal EC_50_ was reported in this paper; accordingly, the original data were re-measured and re-plotted (figure 3*b*) to obtain an EC_50_ of 1.6 × 10^−8^ M. These two values do not differ significantly (*p* = 0.83). Therefore, the concentration dependence of tyramine-induced elevation of intracellular calcium is exactly compatible with an action on chloride shunt conductance.

**Figure 4.**
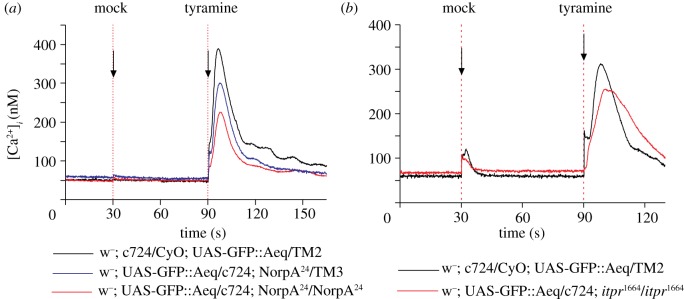
Like *Drosophila* kinin, tyramine calcium signalling is mediated by phospholipase C and the IP_3_ receptor. (*a*) Comparison of calcium responses in lines carrying 2, 1 or 0 copies of *NorpA*, the major phospholipase C of tubules. Typical traces. (*b*) Comparison of *itpr^1664^/itpr^1664^* homozygous mutant flies with wild type. Note that, because of extensive pupal lethality of *itpr* mutants, these experiments were performed on feeding third instar larvae. Each trace is the average of three independent replicates.

Both *Drosophila* kinin and tyramine signal through distinct GPCRs (*lkr* and *CG7431*, respectively [24,37,38]), but use the same downstream messenger. It was therefore of interest to establish whether tyramine signals through phospholipase C (PLC) and inositol trisphosphate (IP_3_), as has previously been established for *Drosophila* kinin [39]. This was tested using well-known mutants for the widely expressed PLC, *no receptor potential A* (*norpA*), and for the only InsP_3_ receptor gene, *itpr. NorpA* nulls are viable, because there is a second PLC in *Drosophila* (*Plc21C*), so using the null *norpA^24^* it was possible to study the calcium response in tubules with 2, 1 or 0 working copies of *norpA* (figure 4*a*). As can be seen, reduction in the number of copies of *norpA* produced a corresponding reduction in calcium response, as previously shown for the neuropeptide *Drosophila* kinin [39]. PLC acts to liberate InsP_3_, which classically acts on its cognate receptor in the endoplasmic reticulum to produce a rapid calcium pulse, which typically triggers further calcium entry into the cell. As *iptr* is a single copy gene in *Drosophila*, nulls are lethal [40]—perhaps surprisingly as late as the pupal stage—and so the impact of *itpr* was assessed in feeding third instar larvae (figure 4*b*). As can be seen, in *itpr^1664^/itpr^1664^* hypomorphs, the calcium response was attenuated. Therefore, although the tyramine and *Drosophila* kinin signals originate from different sources and act on distinct receptors, their downstream signalling through NorpA, Itpr and Ca^2+^_i_ is indistinguishable.

**Figure 5.**
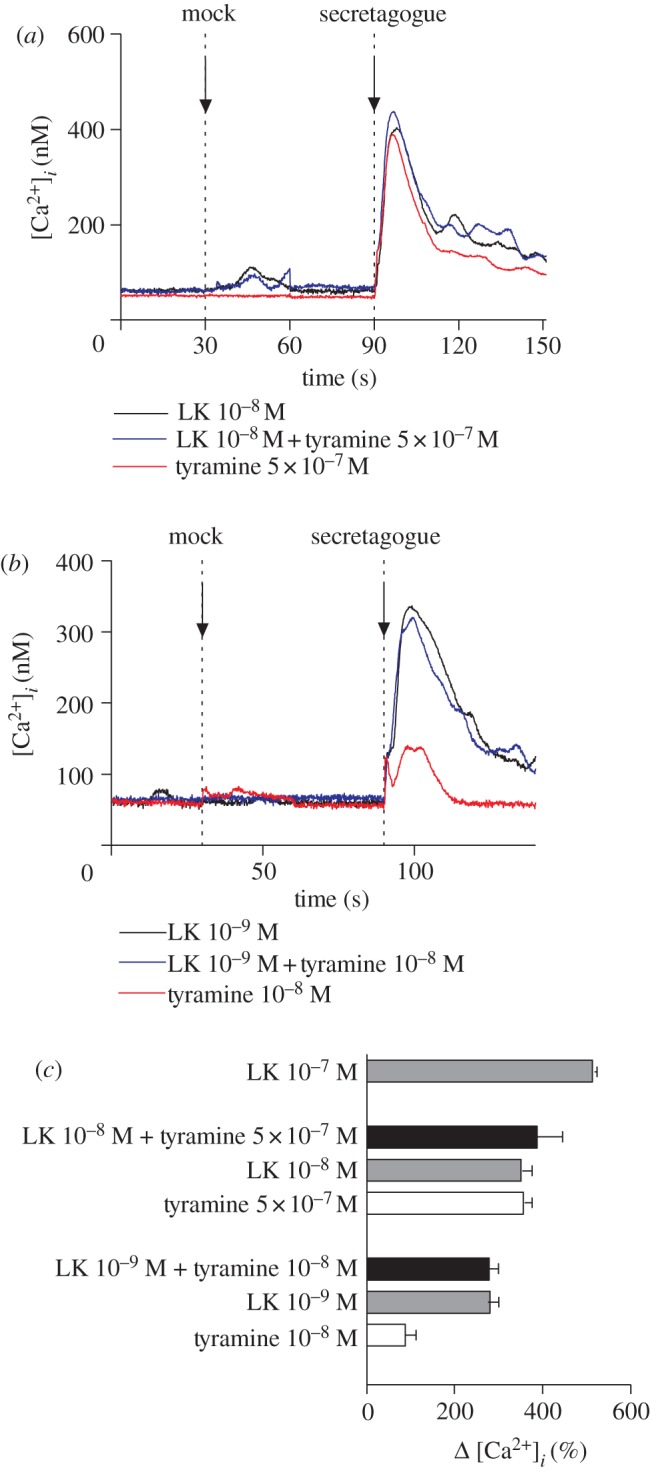
Tyramine calcium signalling in stellate cells is not synergistic to that of *Drosophila* kinin. (*a*) Traces from experiment with high concentrations of kinin and tyramine. (*b*) Traces from experiment with lower concentrations of kinin and tyramine. (*c*) Peak increases in calcium signals (relative to basal) observed in A and B, with a saturating concentration of kinin (10^−7^ M) for reference. Tubules from adult c724>GFP::aeq flies were dissected and exposed to *Drosophila* kinin, tyramine or both at the point indicated, and responses compared with mock injections at 30 s. Typical traces.

Is parallel activation of the *Drosophila* kinin and tyramine pathways synergistic? As both act through the same second messenger, this would not be expected; and indeed (figure 5), the calcium response to tyramine and *Drosophila* kinin combined is not significantly greater than to either secretagogue separately, at either high or submaximal concentrations of the two agonists. Indeed, there is little evidence for additivity in the signals, implying that the two pathways converge on a limiting downstream component.

**Figure 6.**
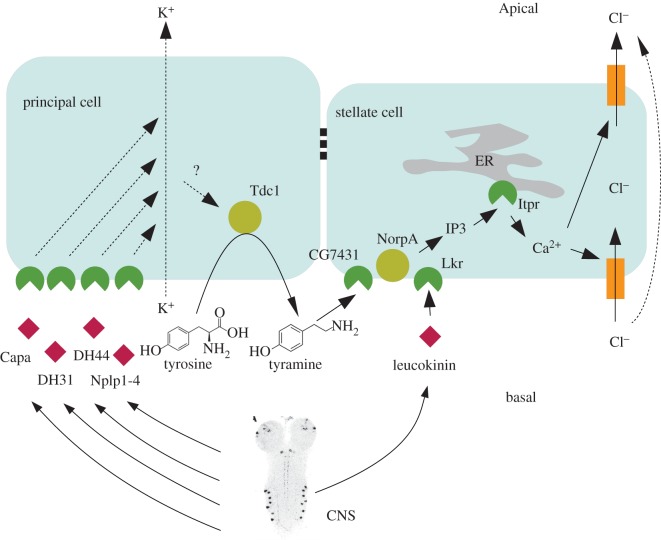
A possible model for the parallel activation of the stellate cell by tyramine and *Drosophila* kinin. Neuropeptides (red diamonds) from the CNS, or neuroendocrine cells in the gut, activate electrogenic cation pumping in the principal cell, or shunt conductance in the stellate cell. As tyrosine decarboxylase 1 is located in the principal cell, there is also the potential for principal cell secretagogues to influence Tdc1 activity, and thence tyramine production. The image of the CNS (in fact stained for *Drosophila* kinin) is taken from [41].

Overall, then, an intriguing model has been demonstrated, in which two distinct secretagogues with two different origins within the organism elicit responses which are indistinguishable downstream, with both acting through PLC and InsP_3_ to elevate intracellular calcium, and thence to trigger a massive and rapid increase in the chloride shunt conductance. At first sight, such a system would seem to defy Occam's razor; why should such independent pathways exist? The solution proposed by Blumenthal [28] is based on the origins of the two signals (figure 6). *Drosophila* kinin is a *bona fide* neuropeptide, which has been mapped to neurosecretory cells in the CNS and peripheral tissues [42–45]. It thus provides a clear route through which the CNS controls diuresis. In contrast, tyramine is generated from tyrosine by the action of tyrosine decarboxylase, which is found in the adjacent principal cells within the tubule itself [28]. The principal cells are themselves under neuroendocrine control, from both the CNS and neurosecretory cells in the midgut [46], and are the sites for active cation transport. The parallel activation model would thus allow the cation pumping cell (which sets up the TEP gradient for chloride) to influence the conductance of the chloride shunt pathway directly, and so produce efficient diuresis. So the potential exists for neuroendocrine stimulation of the principal cell, by any of the neuropeptides DH_31_, DH_44_, CAPA or Nplp1-4, to not only increase the driving force for chloride (by pumping cations to the lumen), but also to increase the conductance for chloride simultaneously. While further work is needed, such a mechanism would be parsimonious, as increasing active transport of cations without increasing the chloride shunt conductance necessary for fluid secretion would be energetically wasteful. With two secretagogues with very different threshold concentrations, there is also the scope to tune the system over a broad range of inputs.

This pathway should be seen in the context of multiple opportunities for cross-talk in the control of the insect renal system. For example, although central control of renal function is widely studied, there are neurosecretory cells in the midgut which contain—and so may co-release—several pairs of neuropeptides that are known to act on the tubule; for example, kinin and DH_31_, or short neuropeptide F and DH_31_ [46]. In *Locusta* [47] and *Rhodnius* [48], the DH_44_ and kinin homologues co-localize in the same abdominal neurosecretory cells. Within the CNS, the *Drosophila* kinin receptor is known to be expressed on the neurosecretory cells that express DH_44_ [18,24]. In small animals, scaling arguments suggest that ion and water homeostasis are critical for survival, so perhaps it is not surprising that such a complex network of signals can interact to optimize the response of the renal tubule from moment to moment.
